# Substrates of the m^6^A demethylase FTO: FTO-LINE1 RNA axis regulates chromatin state in mESCs

**DOI:** 10.1038/s41392-022-01085-w

**Published:** 2022-07-06

**Authors:** Pia Sommerkamp

**Affiliations:** European Molecular Biology Laboratory (EMBL), Meyerhofstraße 1, 69117 Heidelberg, Germany

**Keywords:** Embryonic stem cells, Pluripotency

In a recent paper published in *Science*, Wei et al. report that long-interspersed element-1 (LINE1) RNA is a key target of the N^6^-methyladenosine (m^6^A) ‘eraser’ fat mass and obesity-associated protein (FTO). The FTO-LINE1 RNA axis is involved in chromatin regulation and is essential for mammalian development.^1^

m^6^A is the most common RNA modification in mammalian cells and controls, among others, translation, decay and even chromatin state.^2,3^ The m^6^A landscape is shaped by m^6^A ‘writers’ and ‘erasers’, while the effect of the m^6^A modification is determined by ‘readers’. FTO is an alpha-ketoglutarate (α-KG)-dependent oxygenase, which functions as an m^6^A demethylase (‘eraser’), making m^6^A modifications reversible.^4^ In addition to m^6^A, FTO also mediates demethylation of N6,2′-O-dimethyladenosine (m^6^A_m_) and N1-methyladenosine (m^1^A).^2^ Recent studies have demonstrated an important role for m^6^A in regulating mouse embryonic stem cell (mESC) fate and controlling early mammalian embryonic development.^2^ Wei et al. extend these findings by using *Fto*^*−/−*^ mESCs and mice to identify physiological substrates of FTO and to investigate its role in early mammalian development.^1^

In *Fto*^*−/−*^ mESCs, Wei et al. observed changes in m^6^A levels of chromatin-associated (caRNA) and soluble nuclear fraction RNA, suggesting nuclear FTO substrates. caRNAs, and especially chromosome associated-regulatory RNAs (carRNAs), have recently been shown by the He lab to be targets of the m^6^A ‘writer’ METTL3 in mESCs.^5^ As m^6^A ‘writers’ and ‘erasers’ work together to create an equilibrium of m^6^A modifications, subsets of carRNAs could also represent FTO substrates. In line, Wei et al. observed hypermethylation of carRNAs in *Fto*^*−/−*^ mESCs. The carRNA subset ‘repeat RNAs’ exhibited the strongest correlation between m^6^A hypermethylation and transcript downregulation, with the repeat RNA LINE1 being most strongly affected. LINE1 elements belong to the LINE family and represent autonomous, active retrotransposons, and LINE1 RNA has been shown to regulate chromatin.^6^ Wei et al. observed LINE1 RNA-chromatin interaction as well as co-localization and binding of LINE1 RNA and FTO in mESCs, supporting LINE1 RNA as a physiological target of FTO.

Interestingly, LINE1 RNA is essential for mESCs and is involved in suppression of the 2-cell (2C) program.^3^ 2C-like cells share features of the 2-cell state embryo and are characterized by expression of retrotransposons from the murine endogenous retrovirus with leucine tRNA primer (MERVL) family and repression of ESC pluripotency genes.^3^ Wei et al. observed that *Fto*^*−/−*^ mESCs partially recapitulate this phenotype and exhibit a genetic and phenotypic 2C-like state.

The m^6^A ‘reader’ YTHDC1 has previously been shown to destabilize m^6^A-containing carRNAs.^5^ In line, mechanistic analyses revealed increased binding of LINE1 RNA by YTHDC1 in *Fto*^*−/−*^ mESCs. LINE1 RNA was upregulated upon loss of *Ythdc1*, and *Fto* knockout (KO) led to enhanced LINE1 RNA decay. These results support the notion that an increase in m^6^A modifications of LINE1 RNA could lead to destabilization via YTHDC1 binding. *Fto* KO reduced levels of LINE1 RNA-DNA association and R-loop formation at LINE1 loci. In addition, *Fto*^*−/−*^ mESCs showed decreased RNA synthesis and chromatin accessibility. Closed regions were enriched at m^6^A-modified LINE1 RNA loci. LINE1-containing genes exhibited a stronger decrease in expression upon *Fto* KO, which was accompanied by an increase in intragenic LINE1 RNA m^6^A levels, respectively. Genes containing downregulated intragenic LINE1 RNA were particularly affected, arguing for a *cis*-regulatory role of intragenic LINE1. In line, these gene loci, including early development and pluripotency genes, exhibited a more closed chromatin state. In contrast, 2C genes, such as *Dub1* and *Zscan4*, do not contain LINE1 RNA and exhibited a decrease in expression upon LINE1 RNA-targeted delivery of WT FTO in *Fto*^*−/−*^ mESCs. This indicates that 2 C genes are repressed by LINE1 RNA in the WT setting. Overall, Wei et al. show that LINE1 RNA regulates pluripotency genes in mESCs in *cis*, while 2C genes are regulated in *trans*. This program seems to be governed by demethylation via FTO and installed by regulating chromatin accessibility. *Fto* KO, in contrast, induces closed chromatin at pluripotency gene sites and releases the repression at 2C genes, leading to the observed 2C-like state and differentiation defects.

In vivo analysis revealed ovarian defects and impaired fertility in female KO mice, i.e. reduced numbers of immature germinal vesicle (GV) oocytes and impaired maturation to the mature MII oocyte stage. GV and MII oocytes exhibited reduced LINE1 RNA levels, more closed chromatin and downregulation of LINE1 RNA-containing genes. Mating of female and male WT and *Fto*^*−/−*^ mice led to genotype-specific phenotypes during embryonic development, with FTO^mat KO/pat KO^ embryos not being viable. Analysis of the *Fto*^mat KO/pat KO^ vs WT morula stage revealed repressed LINE1 RNA, decreased expression of essential regulators of early embryonic development and upregulation of 2C markers. Of note, *Fto*^*−/−*^ offspring (F1) of heterozygous *Fto*^*+/−*^ mice (P) is viable. The studied phenotypes were observed in the F2 generation after intracytoplasmic sperm injection using female and male *Fto*^*−/−*^ animals.

In the future, it will be important to further explore the mechanistic link between the FTO-LINE1 axis and YTHDC1. Does FTO demethylate LINE1 RNA in mESCs and during embryonic development to specifically prevent YTHDC1 binding and subsequent LINE1 RNA destabilization and chromatin closure? What other factors are involved in this process, and how are the different functions of LINE1 RNA in *cis* and *trans* achieved? To answer these questions, it will be useful to investigate additional substrates of FTO, including other repeat RNAs identified in this study. In addition, it will be important to understand how the cell type-specific function of FTO is achieved, especially cytosolic versus nuclear function. This and previous studies have highlighted the importance of the m^6^A landscape for mediating chromatin-regulatory functions of RNA.^3^ m^6^A ‘readers’ seem to be important in mediating this crosstalk and are likely to interact with protein complexes involved in DNA methylation and histone modification. But how exactly the FTO-LINE1 RNA axis is involved in these processes remains to be determined. Interestingly, regulation of transposable elements emerges as one of the major targets of this m^6^A RNA-chromatin axis and plays a role in development. Repeat RNAs, such as LINE1, seem to be highly important to suppress the 2C-like state in mESCs, as shown by Wei et al. and others.^3^ Interestingly, the 2C-like state is characterized by the expression of MERVL retrotransposon family members. Different retrotransposons are transcribed during embryogenesis, and this pattern seems to partially be controlled by m^6^A methylation of repeat RNAs, which in turn seem to control chromatin accessibility.

**Fig. 1:**
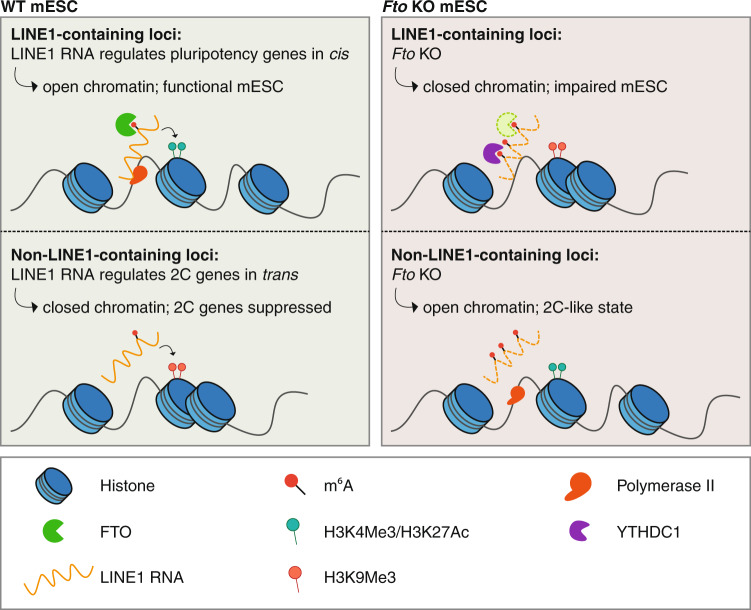
Schematic representation of the function of LINE1 RNA in mESCs and consequences of *Fto* KO. LINE1 RNA is the main substrate of FTO in mESCs. In WT mESCs, LINE1 RNA promotes an open chromatin state at LINE1-containing gene loci, for example by recruitment of histone modifiers that install activation marks. Mechanistically, loss of FTO leads to an increase in m^6^A levels of LINE1 RNA, which causes destabilization and thereby reduced levels of LINE1 RNA, potentially via YTHDC1. The absence of LINE1 RNA and LINE1 RNA-DNA interaction affects the chromatin state and leads to closed chromatin, installation of repressive histone marks and reduced transcription. This phenomenon is especially observed at the site of LINE1-containing gene loci, which also encode pluripotency genes. These genes are regulated by LINE1 RNA in *cis*, leading to a decrease in expression upon *Fto* KO. LINE1 has been shown to be important for silencing of 2C genes.^3^ Interestingly, a release of repression of non-LINE1-containing 2C genes is observed in *Fto*^*−/−*^ mESCs, suggesting a potential role for LINE1 RNA in regulating these loci in *trans*. Functionally, this leads to a more 2C-like state and loss of the mESC state, including reduced expression of several pluripotency genes and impaired differentiation and self-renewal

In summary, Wei et al. could show that LINE1 RNA is the major target of FTO in mESCs (Fig. 1). Activity of FTO in the nucleus ensures LINE1 RNA demethylation, which is important for the accessibility and transcription of LINE1-containing genes, including pluripotency genes. This process is mediated by LINE1 RNA in *cis*. In contrast, 2C genes are suppressed by FTO, presumably via LINE1 RNA-mediated regulation in *trans*. This novel FTO-LINE1 axis is also essential for oocyte and embryo development.
